# Identification of hypoxia-related genes and exploration of their relationship with immune cells in ischemic stroke

**DOI:** 10.1038/s41598-023-37753-2

**Published:** 2023-06-29

**Authors:** Kai Yang, Zhaoqi Zhang, Xiaoju Liu, Tong Wang, Zhicheng Jia, Xin Li, Wei Liu

**Affiliations:** 1 Acupuncture and Moxibustion and Massage College, Shandong University of Traditional Chinese Medicine, Jinan, China; 2 Shandong University of Traditional Chinese Medicine, Jinan, China; 3 Department of Neurology, The Affiliated Hospital of Shandong University of Traditional Chinese Medicine, Jinan, China; 4 Department of Cerebral Disease, The Second Affiliated Hospital of Shandong University of Traditional Chinese Medicine, Jinan, China

**Keywords:** Neuroscience, Immunology

## Abstract

Ischemic stroke (IS) is a major threat to human health, and it is the second leading cause of long-term disability and death in the world. Impaired cerebral perfusion leads to acute hypoxia and glucose deficiency, which in turn induces a stroke cascade response that ultimately leads to cell death. Screening and identifying hypoxia-related genes (HRGs) and therapeutic targets is important for neuroprotection before and during brain recanalization to protect against injury and extend the time window to further improve functional outcomes before pharmacological and mechanical thrombolysis. First, we downloaded the GSE16561 and GSE58294 datasets from the NCBI GEO database. Bioinformatics analysis of the GSE16561 dataset using the limma package identified differentially expressed genes (DEGs) in ischemic stroke using adj. p. values < 0.05 and a fold change of 0.5 as thresholds. The Molecular Signature database and Genecards database were pooled to obtain hypoxia-related genes. 19 HRGs associated with ischemic stroke were obtained after taking the intersection. LASSO regression and multivariate logistic regression were applied to identify critical biomarkers with independent diagnostic values. ROC curves were constructed to validate their diagnostic efficacy. We used CIBERSORT to analyze the differences in the immune microenvironment between IS patients and controls. Finally, we investigated the correlation between HRGs and infiltrating immune cells to understand molecular immune mechanisms better. Our study analyzed the role of HRGs in ischemic stroke. Nineteen hypoxia-related genes were obtained. Enrichment analysis showed that 19 HRGs were involved in response to hypoxia, HIF-1 signaling pathway, autophagy, autophagy of mitochondrion, and AMPK signaling pathway. Because of the good diagnostic properties of SLC2A3, we further investigated the function of SLC2A3 and found that it is closely related to immunity. We have also explored the relevance of other critical genes to immune cells. Our findings suggest that hypoxia-related genes play a crucial role in the diversity and complexity of the IS immune microenvironment. Exploring the association between hypoxia-related critical genes and immune cells provides innovative insights into the therapeutic targets for ischemic stroke.

## Introduction

Stroke is a neurological condition that can be fatal and primarily affects older people. Stroke is the second leading cause of disability and death globally, with low and middle-income countries bearing the most tremendous burden of disease^1^. Stroke can be broadly classified into hemorrhagic stroke and ischemic stroke, and the most common type of stroke in clinical practice is ischemic stroke. Arterial occlusion-related ischemic stroke is the leading cause of most strokes, accounting for 87% of stroke cases and nearly half of all deaths^2^. According to the latest statistics, the global ischemic stroke population grew from 2.04 million to 3.29 million between 1990 and 2019 and is predicted to reach 4.9 million by 2030^3^.

When the arterial blockage occurs, the insufficient supply of oxygen and glucose can produce a series of harmful events^4^, including inadequate ATP supply, lactic acidosis, extracellular excitotoxicity, mitochondrial degradation, neuroinflammation, disruption of the blood–brain barrier (BBB), irreversible neuronal cell death. Impaired cerebral perfusion leads to acute hypoxia and glucose deprivation, which directly results in reduced adenosine triphosphate (ATP) synthesis, leading to lactic acidosis and disturbed cellular homeostasis resulting in cellular damage^5^. In addition, ATP deficiency leads to the failure of ATP-dependent ion transport pumps^6^, which induces extracellular excitability and cytotoxic edema^6^. The activation of associated proteases and lipases leads to free radical release and mitochondrial degradation. Hypoxia/reperfusion can also cause neuronal damage, while damaged neurons can activate resident microglia to move to the infarct core and penumbra^5^, with different subtypes of microglia exerting protective and damaging effects^7^. In addition to this, hypoxia enhances the expression of transcription factors, which in turn promote the synthesis of inflammatory proteins, amplify neuroinflammation, and promote infiltration of peripheral immune cells^5^. Invading neutrophils secrete matrix metalloproteinases, leading to disruption of tight junctions and disruption of the BBB, which in turn exacerbates brain edema, inducing cell death and neuronal loss^5,8^.

Studies have shown that mortality and recurrence rates of ischemic stroke increase with increasing duration of hypoxia^9,10^. Early embolization and pharmacological thrombolysis shorten reperfusion time to improve hypoxia, possibly reducing complications, disability, and mortality^11^. Intravenous systemic thrombolysis with fibrinolytic drugs, mainly alteplase (rt-PA), is the standard of care in acute ischemic stroke. Rt-PA converts fibrinogen into the protein hydrolase fibrinolytic enzyme that cleaves fibrinogen and fibrin. Fibrinolysis leads to the breakdown of blood clots, allowing blood flow to return to the brain^12^. However, the numerous contraindications and narrow time window for intravenous thrombolysis prevent many patients from receiving thrombolytic therapy^13,14^.

Hypoxia plays a vital role in ischemic stroke, which runs through the occurrence and development of ischemic stroke. Based on the fact that a large proportion of the population cannot receive thrombolytic therapy in the prescribed time window. Finding targets related to hypoxia is essential to protect brain cells from ischemia and reperfusion injury and expand the treatment time window.

This study first identified differentially expressed hypoxia-related genes (HRGs) between IS and control samples from metadata cohorts and hypoxia-related datasets. It is helpful to clarify the signal pathway mechanism related to hypoxia and find effective targets for treating IS. Secondly, after integrated analysis among least absolute shrinkage and selection operator (LASSO) regression and multivariate logistic regression, we included critical genes in the receiver operating characteristic (ROC) curve and compared their diagnostic power for IS. Importantly, we explored the relationship between hypoxia-related genes and immune cells, providing a new perspective for developing immunomodulatory therapeutic options for IS.

## Methods

### Dataset preparation and data processing

Transcriptome profiles of IS patients containing the messenger RNA (mRNA) expression profiles and clinical information were obtained from Gene Expression Omnibus (GEO)database (https://www.ncbi.nlm.nih.gov/geo) on 11 August 2022. We included whole or peripheral blood gene expression profiles from patients with IS or control samples. This study used R to download ischemic stroke-related mRNA expression profile data and clinical data from GEO database.

The GSE16561^15^ dataset (GPL6883, Illumina HumanRef-8 v3.0 expression bead chip, array, Homo sapiens) contained 39 control subjects and 24 patients with ischemic stroke, serving as the training set. The GSE58294^16^ dataset (GPL570, Affymetrix Human Genome U133 Plus 2.0 Array, Homo sapiens) contains a total of 23 control samples and 69 ischemic stroke samples blood samples from less than 3 h, 5 h, and 24 h after the onset of ischemic stroke, serving as a validation cohort. More details about these two datasets are shown in Table 1.

**Table 1:** Detailed information of the studied gene expression profiles.

Dataset	Platform	Control	IS	Author	Country	Submission	Sample	Application
GSE16561	GPL6883	24	39	Barr TL	USA	2010	Peripheral whole blood	Identification
GSE58294	GPL570	23	69	Stamova B	USA	2014	Peripheral whole blood	Validation

In addition, 259 hypoxia-related genes were obtained according to the Molecular Signature database^17^ and Genecards (https://www.genecards.org/). There were 200 hypoxia-related genes in the Molecular Signature database. In the Genecards database, we searched for hypoxia-related genes using "hypoxia" as the search term and obtained 68 genes with an association score > 7 as the cut-off value. After removing overlapping genes, 259 HRGs were obtained (Supplementary Table 1).

### Differential expression analysis

Differentially expressed genes (DEGs) between ischemic stroke and control samples in the GSE16561 were identified by the package of limma in R language (adj. p. values < 0.05 and |log2FC| ≥ 0.5)^18,19^. DEGs overlapped with hypoxia-related genes were regarded as differentially expressed hypoxia-related genes (HRGs). Meanwhile, the volcano map and cluster heatmap were represented by the package "pheatmap (v1.0.12)" and "ggplot2(v3.3.6)" in R to visualize the differences.

### Functional enrichment analysis

Biological functions were analyzed using the clusterProfiler package (v4.4.4), which includes GO and KEGG. The above HRGs were comprehensively investigated using the MF, CC, BP, and KEGG, which refer to molecular function, biological process, cellular component, and the Kyoto Encyclopedia of Genes and Genomics pathway (v97.0)^20,21^. We used the adjust the error discovery rate FDR to represent significant results. P < 0.05 as the screening criterion.

### Identification of diagnostic markers and construction of the diagnosis model

Minimum absolute shrinkage selection operator (LASSO) logistic regression was used to reduce the number of genes in the model and solve the multicollinearity problem in regression analysis. The LASSO algorithm was applied using the "glmnet" package^22^ to determine feature selection and screening of IS diagnostic markers. We then used multivariate logistic regression and inverse methods to identify independent diagnostic biomarkers.

### Diagnostic model internal and external validation

The "pROC" (v1.18.0) package^23^ was used to access the diagnostic performance of selected HRG biomarkers in the training dataset (GSE16561). We then validated the diagnostic performance of crucial biomarkers that differentiate IS patients from controls in an external validation cohort (GSE58294). To assess the reliability and generalizability of the genetic characteristics, we selected the GSE58294 dataset containing samples from different time points within 24 h after the onset of ischemic stroke as the validation set. We evaluated the diagnostic model using the same parameters.

### Immune infiltration analyses

At the same time, to compare the relative percentages of immune cells in each sample, CIBERSORT was used based on the mRNA expression^24^. Herein, we performed "e1071 (v1.7-11)", "parallel" (v4.2.0), "preprocess Core (v1.48.0)" and "CIBERSORT" packages in R to evaluate. The bar graph provides an overview of each individual's relative percentage of the 22 immune cell subsets. The associations of all cell subsets in the form of correlated heat maps were shown by the corrplot (v0.90) package in R. And the differences in infiltration between IS and control samples were reflected in the form of violin maps using the ggplot2(v3.3.0) package. The critical value was P < 0.05.

### Correlation analysis between key HRGs and immune cells

Spearman correlation analysis was then performed for key diagnostic biomarkers and immune cell subsets in IS to estimate their relationship using the "ggstatsplot(v0.9.3)" package in R. Meanwhile, the results of the correlational analysis was visualized by the "tidyverse (v1.3.2)", "ggsci(v2.9)" and "ggplot2 (v3.3.6)" packages in R.

### Statistical analyses

Statistical analysis and drawing work were performed using R software (version 4.2.0). The ROC analysis was then visualized using the R's "pROC" package (v1.18.0). Continuous variables were represented by mean ± SD, Student's t-test represented normal distribution variables, abnormal distribution variables were represented by Mann–Whitney U test. The cut-off thresholds of adj.p.value < 0.05 and |log2FC|≥ 0.5 were established as the cut-off thresholds in the differential expression analysis. Of all the studies, we considered P < 0.05 as a meaningful difference.

## Result

### Identification of 19 differentially expressed HRGs

**Figure 1:**
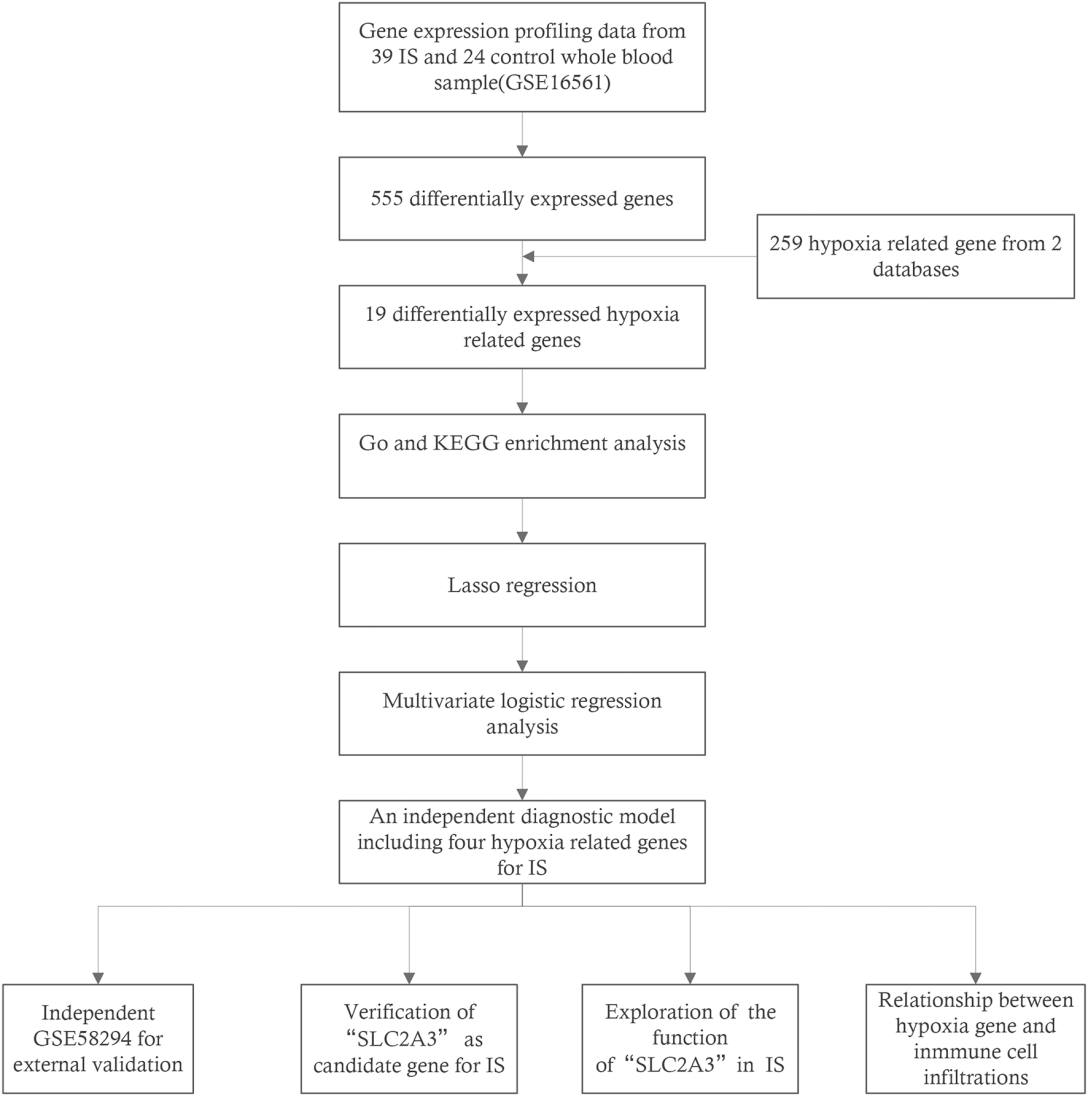
The flowchart of the overall study procedures.

**Figure 2:**
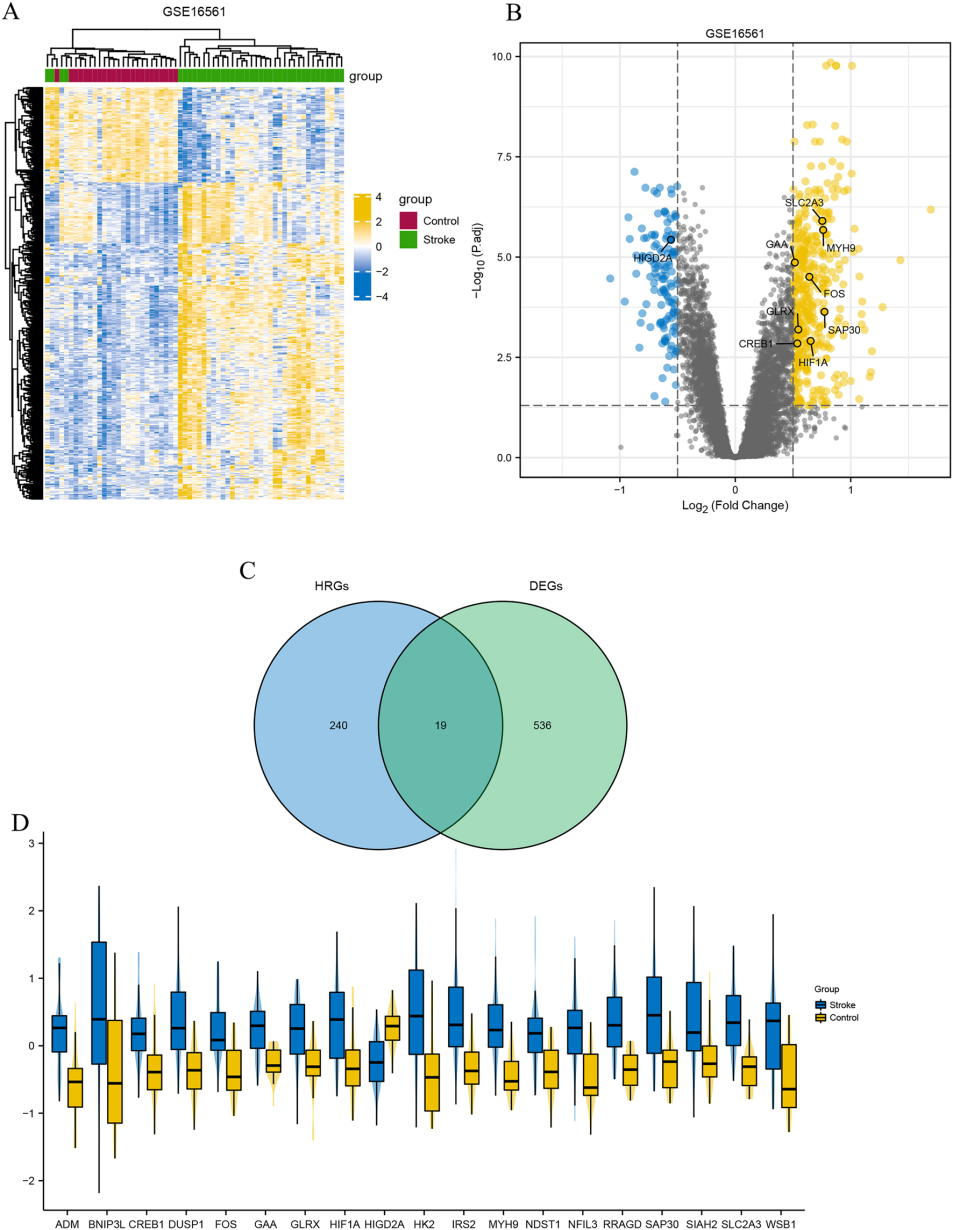
Identification of DEGs and hypoxia-related DEGs. (**A**) The heatmap of DEGs in GSE16561. (**B**) Volcano map of GSE16561. (**C**) Venn diagrams show the overlap between the significant differentially expressed genes and hypoxia-related genes. (**D**) Boxplots of the expression levels of 19 differentially expressed HRGs in IS and healthy controls. The blue box plots above the corresponding gene name represent the expression in IS, whereas the yellow box plots represent the expression in healthy controls.

The flow chart in Fig. 1 shows an overview of this study. The data for analysis was obtained from the online GEO database. The data of 39 whole IS blood samples and 24 control whole IS blood samples in the GSE16561 dataset were obtained, including gene expression profiles and clinical information, according to the following standards identified 555 DEGs: |logFC| ≥ 0.5 or higher, adj. p. value < 0.05. Four hundred twenty-seven up-regulated genes (76.9%) and 128 down-regulated genes (23.1%) among DEGs were illustrated in the volcano plot (Fig. 2A,B). By intersecting 259 HRGs, Venn diagrams revealed 19 differentially expressed HRGs (HIF1A, HIGD2A, CREB1, FOS, ADM, BNIP3L, DUSP1, GAA, GLRX, HK2, IRS2, MYH9, NDST1, NFIL3, RRAGD, SAP30, SIAH2, SLC2A3, and WSB1) (Fig. 2C). Figure 2D showed the expression of these 19 genes in GSE16561. It can be seen from the data in the picture that, except for HIGD2A, other genes were both up-regulated in the ischemic stroke sample. Additional details about them are provided in Table 2.

**Table 2:** Information on the 19 differentially expressed hypoxia-related genes.

Gene	Full name	logFC	adj.P value
HIF1A	Hypoxia inducible factor 1 subunit alpha	0.652461	0.0012319
HIGD2A	HIG1 hypoxia inducible domain family member 2A	− 0.55777	3.708E−06
CREB1	CAMP responsive element binding protein 1	0.537547	0.0014228
FOS	Fos proto-oncogene, AP-1 transcription factor subunit	0.641672	3.112E−05
ADM	Adrenomedullin	0.837845	8.063E−06
BNIP3L	BCL2 interacting protein 3 like	0.943066	0.0088373
DUSP1	Dual specificity phosphatase 1	0.760383	1.776E−05
GAA	Alpha glucosidase	0.51596	1.367E−05
GLRX	Glutaredoxin	0.545609	0.0006402
HK2	Hexokinase 2	0.869945	0.0006451
IRS2	Insulin receptor substrate 2	0.804123	4.475E−05
MYH9	Myosin heavy chain 9	0.760886	2.106E−06
NDST1	N-deacetylase and N-sulfotransferase 1	0.608013	0.0002353
NFIL3	Nuclear Factor, Interleukin 3 Regulated	0.770197	8.058E−06
RRAGD	Ras related GTP binding D	0.733595	5.264E−06
SAP30	Sin3A associated protein 30	0.772688	0.0002318
SIAH2	Siah E3 ubiquitin protein ligase 2	0.552496	0.0135577
SLC2A3	Solute carrier family 2 member 3	0.753791	1.253E−06
WSB1	WD repeat and SOCS box containing 1	0.72695	0.0005869

### Functional enrichment analysis of 19 HRGs

**Figure 3:**
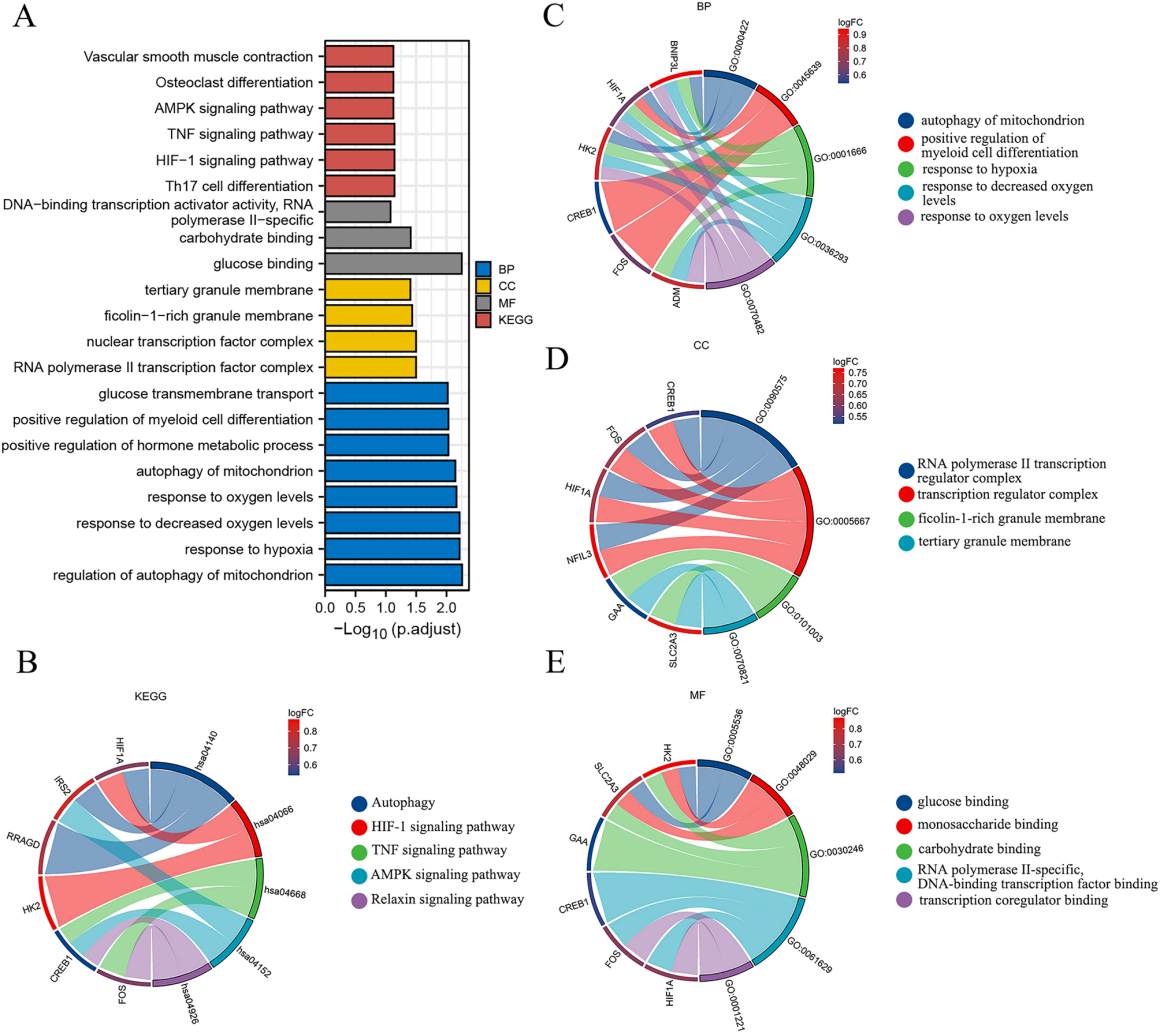
Functional enrichment for HRGs. (**A**,**B**) GO and KEGG analysis of 19 HRGs. (**B**–**E**) The enriched items in GO and KEGG analysis. "BP" stands for "biological process", "CC" stands for "cellular component," "MF" stands for "molecular function," and "KEGG" stands for "Kyoto Encyclopedia of Genes and Genomes".

KEGG enrichment revealed the 19 HRGs predominantly participated in autophagy, HIF-1 signaling pathway, AMPK signaling pathway, TNF signaling pathway, growth hormone synthesis, secretion and action, Th17 cell differentiation, TNF signaling pathway, Relaxin signaling pathway (Fig. 3A,B). BP showed that the selected genes were principally enriched in the regulation of autophagy of mitochondrion, response to hypoxia, response to decreased oxygen levels, response to oxygen levels, and positive regulation of myeloid cell differentiation (Fig. 3C). In CC, HRGs were mainly associated with the RNA polymerase II transcription factor complex, nuclear transcription factor complex, transcription factor complex, and tertiary granule membrane (Fig. 3D). MF showed that 19 HRGs were mainly related to transcription corepressor activity, monosaccharide binding, glucose binding, monosaccharide binding, and RNA polymerase II-specific DNA-binding transcription factor binding (Fig. 3E). The other detailed information is listed in Supplemental Table 2.

### Construction of the diagnostic risk model

**Figure 4:**
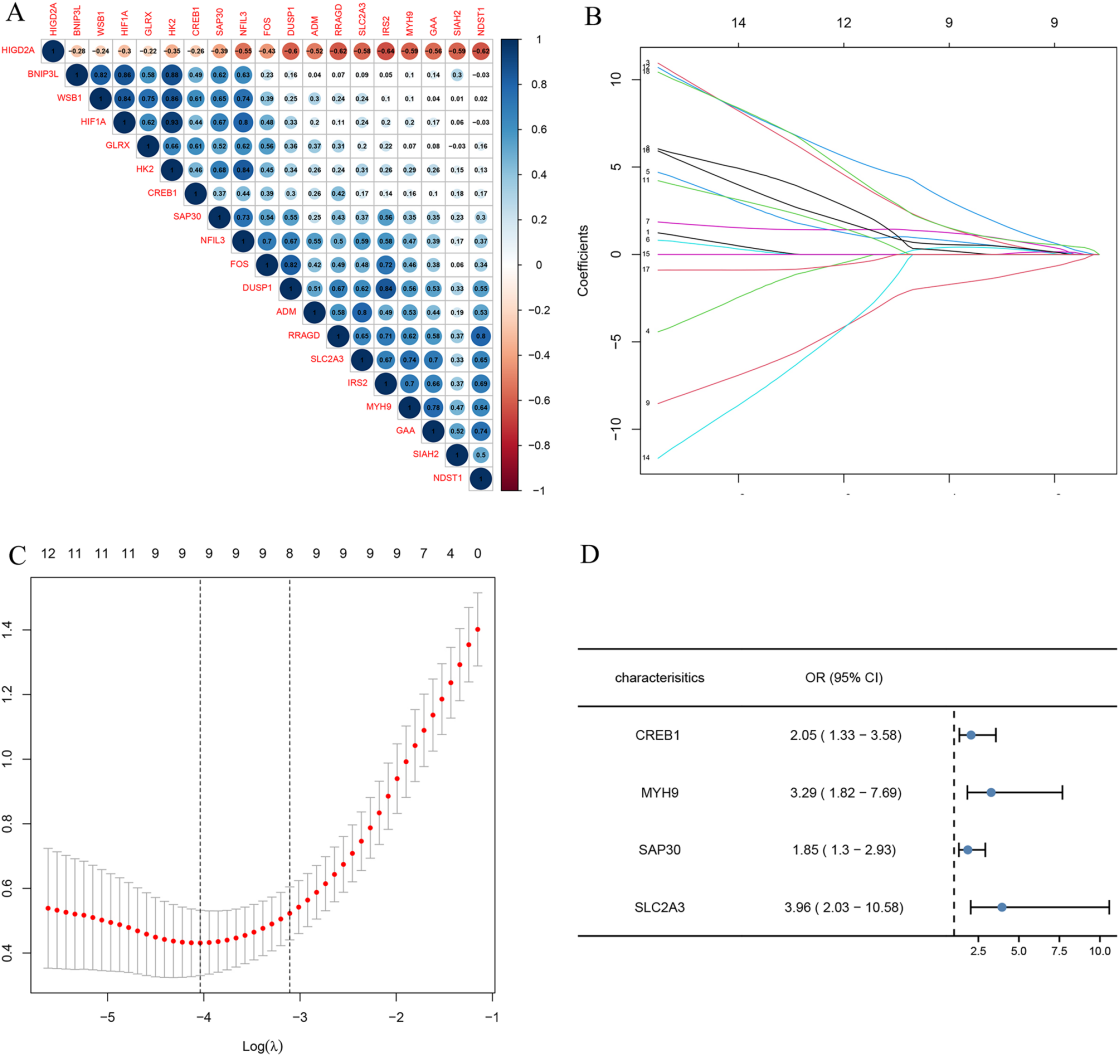
Screening of the optimal HRGs used for the final construction of the diagnostic model. (**A**) Correlation heat map of 19 differentially expressed hypoxia-related genes. The size of the colored squares represents the strength of the correlation; blue represents a positive correlation, and red represents a negative correlation. The darker the color is, the stronger correlation is. (**B**,**C**) Screening of the optimal parameter (using lambda.1se as the best lambda) at which the vertical lines were drawn. LASSO coefficient profiles of the 19 differentially expressed hypoxia-related genes. (**D**) Multivariate logistic regression determined independent candidate diagnostic biomarkers.

**Figure 5:**
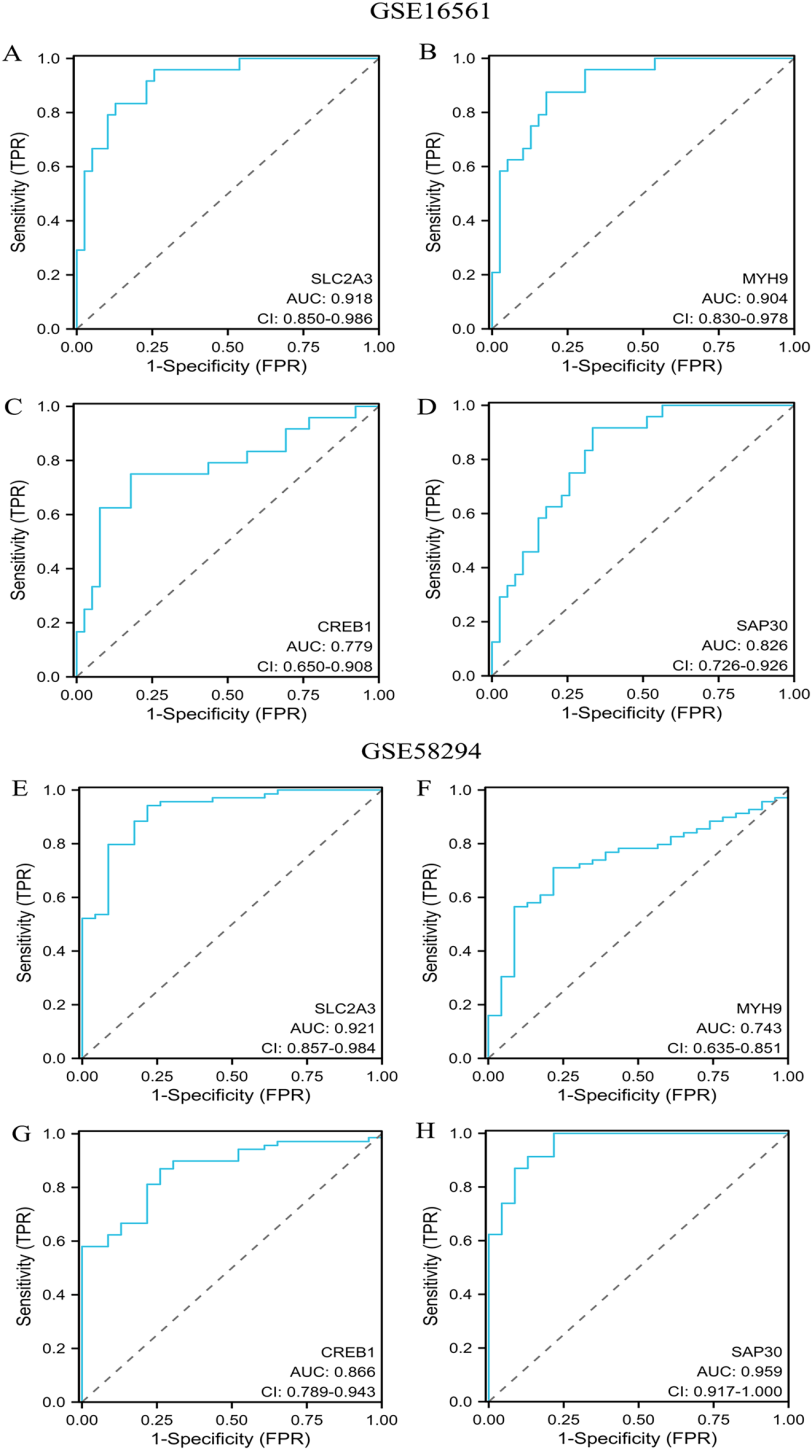
ROC analysis revealed the diagnostic value of hypoxia-related genes in ischemic stroke. (**A**–**D**) The AUC of hypoxia-related biomarkers in the diagnosis of ischemic stroke in ROC analysis in the GSE16561 dataset. (**E**–**H**) The AUC of hypoxia-related biomarkers in the diagnosis of ischemic stroke in ROC analysis in the GSE58294 dataset.

From the graph in Fig. 4A, we can see the correlation matrix analysis of 19 genes, and partly hypoxia genes had a tough correlation with each other. In order to deal with the multicollinearity problem in regression analysis, LASSO analysis was applied to further zoom out the scope of HRGs as possible diagnostic biomarkers for IS. 19 genes were included in the LASSO model. Primarily, 9 genes (CREB1, FOS, GAA, GLRX, HIF1A, HIGD2A, MYH9, SAP30, SLC2A3) were identified based on lambda.1se (Fig. 4B,C). Then, multivariate logistic regression analysis was used to determine the independent candidate diagnostic biomarkers. Four genes (SLC2A3, MYH9, CREB, and SAP30) were obtained (Fig. 4D). Besides, the four biomarkers demonstrated advantageous diagnostic ability and good diagnostic value in discriminating IS from controls. As shown in Fig. 5, the value of SLC2A3, MYH9, CREB1, and SAP30 was 0.918 (95% CI 0.850, 0.986), 0.904 (95% CI 0.830, 0.978),0.779 (95% CI 0.650,0.908), 0.826 (95% CI 0.726, 0.926), respectively.

### External validation of diagnostic model performance

To assess the clinical benefit of the four genes, we used ROC curves to demonstrate their value in discriminating IS diagnoses. And the GSE58294 dataset was used to validate the diagnostic accuracy of the four HRGs biomarkers. As can be seen from the Fig. 5, SLC2A3, MYH9, CREB1 and SAP30 were also indicated admirable diagnostic accuracy with the value were 0.921(95% CI = 0.857, 0.984),0.743(95% CI = 0.635, 0.851),0.866(95% CI = 0.789, 0.943) and 0.959(95% CI = 0.917, 1.000), respectively (Fig. 5).

### SLC2A3 may represent a new candidate gene in IS

**Figure 6:**
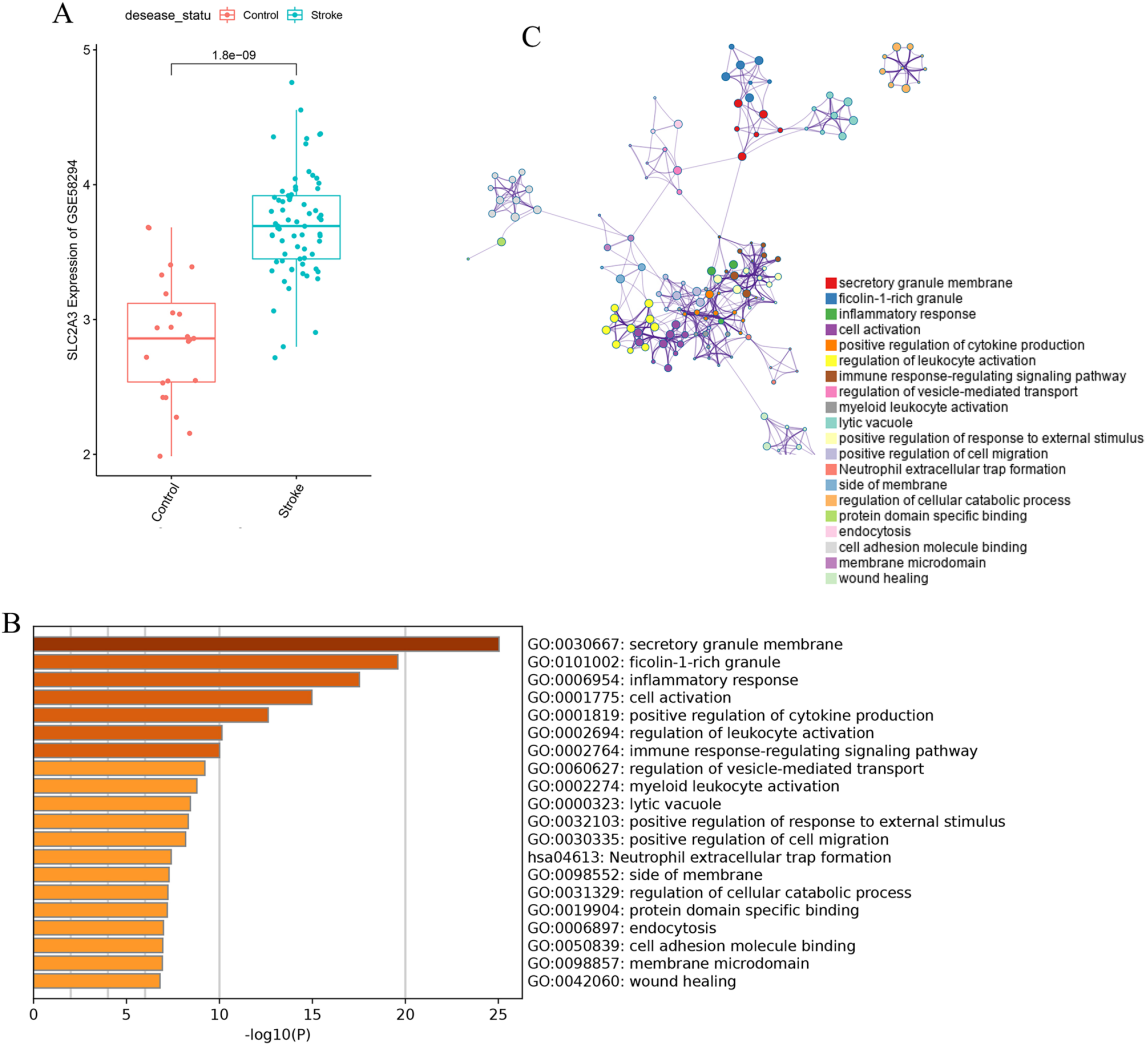
SLC2A3 may represent a new candidate gene in IS. (**A**) SLC2A3 mRNA levels are up-regulated in Ischemic stroke. (**B**,**C**) Enrichment analyses based on GO and KEGG pathways to predict the potential function of SLC2A3 using Metascape.

As Fig. 5 shows, SLC2A3 had the best performance among the four hypoxia genes in distinguishing IS patients from control samples. In order to further detect the character of SLC2A3 in IS, the GSE58294 dataset was analyzed. The SLC2A3 levels in IS patients were expressively higher than those in control samples (Fig. 6A). The results obtained from the preliminary analysis stand up for that SLC2A3 may represent a remarkable candidate gene in IS "Guilt-by-association" method was applied to explore the biological function of SLC2A3 in IS^25^. In order to identify the related genes of SLC2A3, Spearman correlation analysis was performed between SLC2A3 and 555 differentially expressed genes in the patient sample. There were 296 differentially expressed genes that were remarkably associated with SLC2A3 (p < 0.05). In addition, Metascape was applied to explore the functional enrichment analysis. From the chart, it can be seen that these genes were remarkably enriched in Neutrophil degranulation and immune response-regulating signaling pathway, which were closely related to IS^26^ (Fig. 6B,C).

### Immune cell infiltration results

**Figure 7:**
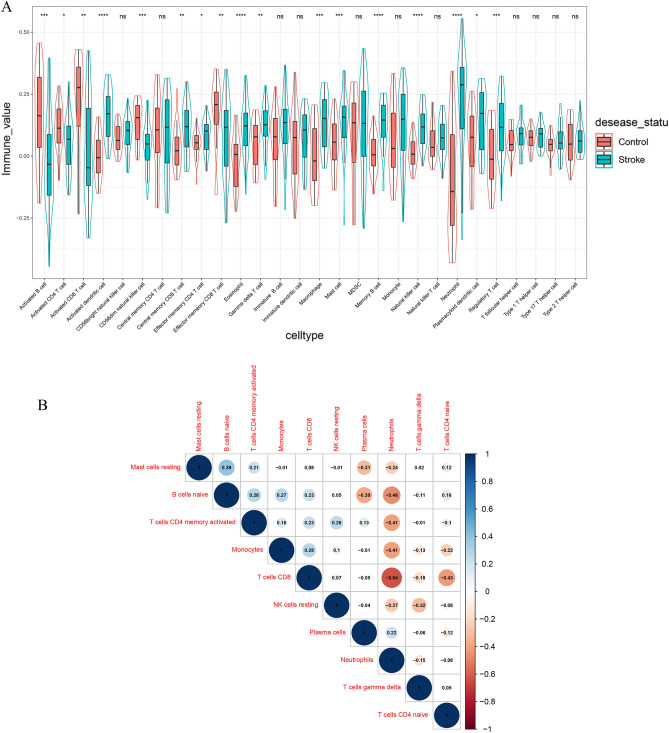
Correlation of infiltrating immune cells. (**A**) The violin plot exhibits the differences in CIBERSOFT immune cell fractions between IS and healthy controls. (**B**) Correlation heat map of immune cells. The size of the colored squares represents the strength of the correlation; blue represents a positive correlation, and red represents a negative correlation. The darker the color is, the stronger correlation is.

The proportions of NK cells, neutrophils, activated dendritic cells, eosinophils, and memory B cells in IS patients were significantly higher than those in control samples. However, the proportion of Activated CD8 T cells and Activated B cells in IS was substantially lower than that in control tissues (Fig. 7A). Correlation heatmap between immune cell subpopulations in ischemic stroke disclosed that Plasma cells were negatively correlated with B cells naive. Neutrophils represented an apparent relationship with T cells CD8, B cells naive, and T cells CD4 memory activated were negatively correlated with T cells CD8. NK cells resting were positively correlated with T cells CD4 memory activated and T cells CD8 (Fig. 7B).

### Relationship between critical HRGs and immune cell

**Figure 8:**
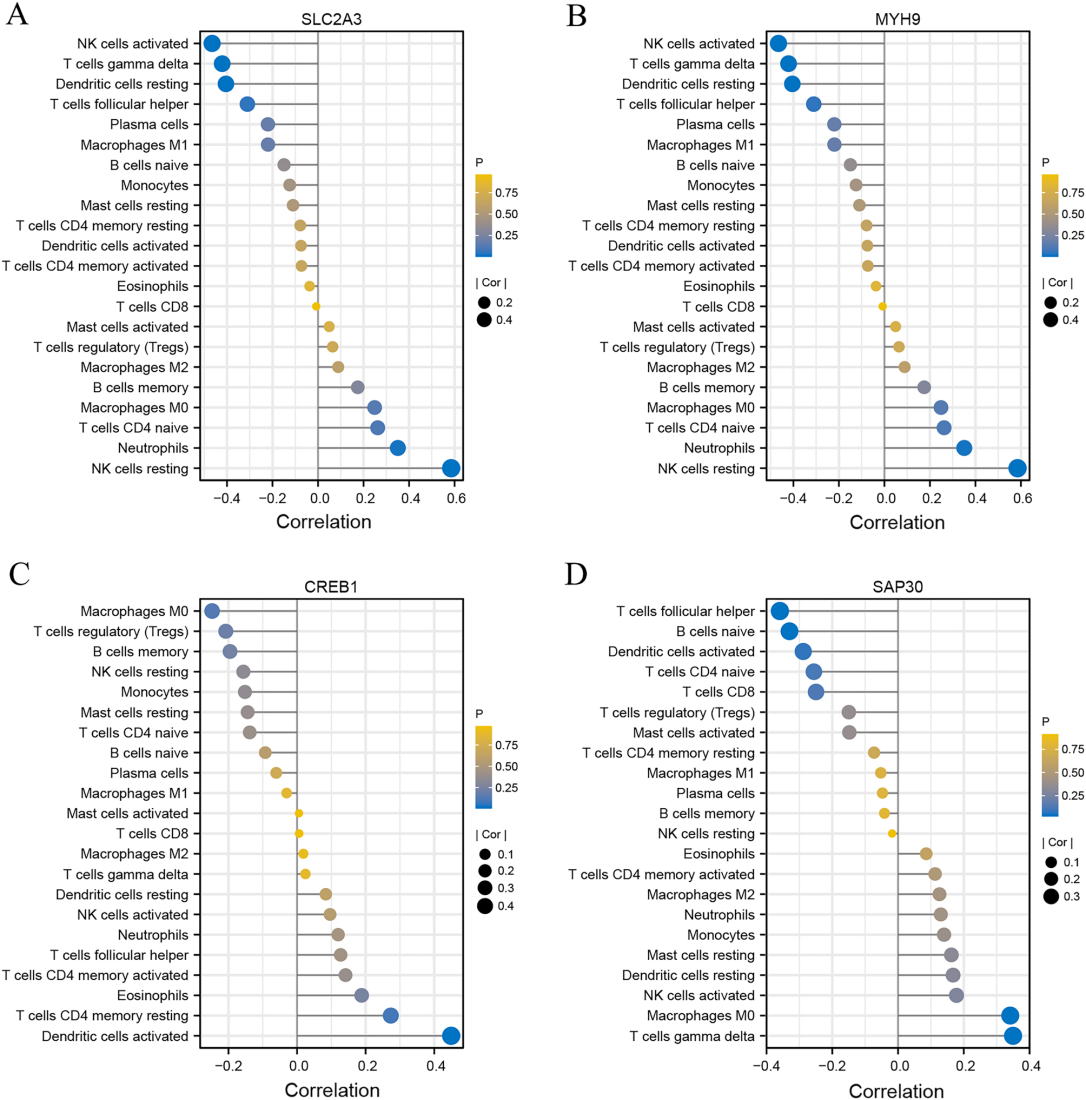
Correlation between CREB1, MYH9, SAP30, and SLC2A3 and immune cells. (**A**) Correlation between SLC2A3 and infiltrating immune cells. (**B**) Correlation between MYH9 and infiltrating immune cells. (**C**) Correlation between CREB1 and infiltrating immune cells. (**D**) Correlation between SLC2A3 and infiltrating immune cells. The size of the dots represents the strength of the correlation between genes and immune cells, the larger dots represent the stronger the correlation, and the smaller dots represent the weaker the correlation.

SLC2A3 had a positive correlation with Neutrophils (r = 0.635, P < 0.001) and Macrophages M0 (r = 0.395, P = 0.012) (Fig. 8A), and MYH9 was not only positively correlated with Neutrophils (r = 0.35, P = 0.026) but also with NK cells resting (r = 0.585, P < 0.001) (Fig. 8B). Besides, CREB1 had a positive correlation with Dendritic cells activated (r = 0.449, P = 0.004) (Fig. 8C). And the Gamma delta T cells was associated with SAP30 (r = 0.350, P = 0.029) (Fig. 8D). These results suggested that the condition of the brain microenvironment in ischemic stroke could be partially reflected by SLC2A3/CREB1/MYH9/SAP30.

## Discussion

Up to now, several reports have shown that HRGs could serve as diagnostic or prognostic markers in many types of tumors, such as osteosarcoma^27^, bladder cancer^28^, and triple-negative breast cancer^29^. However, little research has reported whether hypoxia genes or proteins could be applied as biomarkers in ischemic stroke for early prevention. HRGs play a vital role in the overall development of ischemic stroke. In this study, we have investigated the role of HRGs in the diagnosis of IS. Four HRGs (SLC2A3, CREB1, MYH9, and SAP30) have been identified as crucial biomarkers for IS, and they have established the diagnostic model. External validation analysis also demonstrated that the diagnostic model has an excellent capacity to differentiate IS patients from controls.

We first explored hypoxia-related DEGs in ischemic stroke versus normal controls using GSE16561. As a result, a total of 19 HRGs were identified. In addition, GO and KEGG analysis revealed that these 19 hypoxia-related DEGs were mainly enriched in response to hypoxia, HIF-1 signaling pathway, autophagy, autophagy of mitochondrion, and AMPK signaling pathway. Hypoxia induces peripheral neutrophils to move toward the brain and release proteases, leading to impaired blood–brain barrier integrity and exacerbating oxidative stress^30^. Pericyte HIF-1 can also cause blood–brain barrier disruption while worsening the poor prognosis of stroke^31^. The exact role of autophagy in the pathogenesis of ischemic stroke is controversial. The appropriate autophagy can exert neurological and excessive autophagy leads to neuronal death^32^. Some studies have confirmed that hypoxia and glucose deficiency will activate the AMPK pathway to trigger autophagy. The application of autophagy inhibitors can reduce the area of cerebral ischemia^33^. Interestingly, metformin, as the first-line treatment for diabetes, can play a protective role in ischemic stroke by activating autophagy^34^. Mitochondrial autophagy is a selective autophagy that prevents the production of excess reactive oxygen species (ROS) and subsequent cell death after the onset of ischemic stroke through mitochondrial autophagy^35^.

Immunity plays an essential role in the progression of ischemic stroke, and there are growing studies of neuroprotective effects through neuroinflammatory modulation. The inflammatory mechanisms can exacerbate ischemic tissue damage and worsen clinical outcomes related to excessive immune response^36^. Our results found that the proportions of NK cells, neutrophils, activated dendritic cells, eosinophils, and memory B cells were expressively higher than the control samples. Different immune cells play different roles in ischemic stroke. NK cells can pass the blood–brain barrier and play a dual role in the regulation of the nervous system and immune system.

On the one hand, NK cells exert toxic effects on injured neuronal cells, mediating inflammatory responses involved in cerebral ischemic injury and affecting stroke progression^37^. On the other hand, the injured brain suppresses NK cells-mediated peripheral immune defense to avoid further damage^36^, which will increase susceptibility to infection and cause increased mortality^38^. Peripheral blood neutrophils can be used as an early indicator of stroke outcome. N2 neutrophils also promote macrophage phagocytosis, which is less damaging to ischemic neurons and is expected to be a new treatment^39^. Dendritic cells (DCs) act cooperatively with other immune cells to influence brain injury in ischemic stroke. Animal experiments confirmed that type 2 DCs rapidly infiltrate the brain after MACO and promote neutrophil infiltration by inducing IL-17 production by *γδ* T cells^40^. In addition, DCs initiate Regulatory T cells (Tregs) that suppress immune responses and protect against brain injury caused by MACO^41^.

We applied a machine learning approach for screening essential genes for ischemic stroke using its better predictive performance, lower error rate, and higher reliability^42^. In our study, we screened four signature genes, namely SLC2A3, CREB1, MYH9, and SAP30, by LASSO algorithm and multivariate logistic regression, and these four signature genes have good diagnostic value. These four signature genes had good diagnostic value in the training dataset (GSE16561). Similarly, their diagnostic value results in the validation dataset (GSE58294) were satisfactory. The early prediction and diagnosis of ischemic stroke through diagnostic models are of great importance, especially for patients with a high risk of ischemic strokes, such as TIA^43^, hypertension^44^, and atherosclerosis^45^. Early screening by molecular markers of hypoxia in peripheral blood allows effective stratification of high- and low-risk populations for management, thereby reducing the risk of stroke and improving poor prognosis.

CAMP Responsive Element Binding Protein 1 (CREB1), encoding a transcription factor that belongs to the leucine zipper family of DNA binding proteins. Prior studies have noted that pharmacological intervention can protect ischemic brain tissue by affecting CREB. The application of SMND-309 exerted significant efficacy in protecting neuronal cells from death by triggering the PI3K/Akt/CREB signaling pathway and inducing a considerable increase in BDNF^46^. Improvement of neuroplasticity by interfering with CREB expression has also been reported, resulting in improved disease prognosis^47,48^. CREB1 has multiple roles in neuroimmune. Its phosphorylation inhibits NF-*κ*B activation and suppresses pro-inflammatory responses^49^. At the same time, CREB1 is involved in macrophage generation and differentiation and plays an essential role in myelin regeneration^50,51^.

Myosin heavy chain 9 (MYH9) is one of the members that make up the cytoskeleton^52^. It is involved in blood–brain barrier dysfunction in ischemic stroke^53^ and interacts with actin to mediate oxidative stress-induced neuronal apoptosis^54^. Studies have demonstrated that inhibition or knockdown of MYH9 can slow down the blood–brain barrier damage and exert neuroprotective effects^55,56^. In recent years, attention has also been paid to the vital role of MYH9 in the immune response^57^, which has a positive correlation with NK cells in our study. It promotes cytotoxicity by binding to NK cell lysis granules^58^. In innate immunity, MYH9 has an essential and fundamental role in neutrophil transport, which is closely associated with ischemic stroke^59^. It provides a new biomarker and therapeutic target for hypoxia-induced blood–brain barrier damage.

Sin3A-related protein 30 (SAP30), a gene encoding a protein of the histone deacetylase complex, contains SIN3, SAP18, HDAC1, HDAC2, RbAp46, RbAp48, and other polypeptides. There are few reports on SAP30 in ischemic stroke, while HDAC1 has been reported to play a vital role in neurological diseases. Improving cerebral ischemic neuroinflammation by modulating SAP30 levels and inducing M1 microglia polarization^60^ provides a new therapeutic idea for neuroprotective effects.

SLC2A3 (Solute Carrier Family 2 Member), a member of the Solute Carrier Family, plays a crucial part in the central nervous system. SLC2A3 can be involved in glucose transport across the plasma membrane and blood–brain barrier transport^61^, which is essential for hypoxia and glucose deprivation after the onset of ischemic stroke. The adaptive upregulation of SLC2A3 may slow neuronal apoptosis by increasing glucose transport^62^, thus improving prognosis. In addition, SLC2A3 can affect neuroinflammation after ischemic stroke by influencing the conversion of CD4T cells to Treg cells^63,64^. Based on the adaptive upregulation of SLC2A3 under hypoxia and glucose-deficient conditions, it may be applied to the early diagnosis and target intervention of ischemic stroke.

Since SLC2A3 has the best diagnostic effect, therefore, we further explored the biological role of SLC2A3 in ischemic stroke. Spearman correlation analysis was performed between SLC2A3 and 555 differentially expressed genes in patient samples. And a total of 296 differentially expressed genes were found, and they were significantly associated with SLC2A3 (p < 0.05). Furthermore, we applied the Metascape online tools to conduct the analyses of complete functional and pathway enrichment. These genes associated with SLC2A3 were significantly associated with immune response and immune cell activation and interaction (Fig. 6B). Neuronal damage mitigation by modulation of SLC2A3 may be an effective therapeutic strategy that would benefit individuals who suffer from or are at high risk for ischemic stroke.

It should not be overlooked that this study has several limitations. First, our current study was conducted based on a public dataset with profiles from blood samples rather than brain tissue, which is heterogeneous from the actual immune infiltration in ischemic stroke. Second, the number of published data is limited, and the clinicopathological parameters are not comprehensive, which may lead to potential errors or bias. Third, it is that the training and validation datasets are from different platforms. The effect of differences between platforms on the results cannot be excluded. Fourth, microarray data are not clinically practical, so research and detection of critical markers for immune cells will be an essential part of future work. The last drawback is that the causal relationship between hypoxia-related biomarkers and immune cells and stroke can only be predicted by theoretical analysis rather than prospective studies. Therefore, we will continue to monitor the progress of stroke studies, and further investigation is needed to confirm whether our novel biomarkers and immune cells are potential prognostic predictors or therapeutic targets for stroke.

In conclusion, our studies indicate that the selected four genes may serve as new key biomarkers to assist the diagnosis of ischemic stroke patients from the people at the early stage and serve as new potential therapeutic targets. Meanwhile, it is apparent from the results that immune cells have strong connections with SLC2A3, CREB1, MYH9, and SAP30, which may serve as the new therapeutic target. More biological and clinical experiments are needed to confirm further the function of the selected genes and immune cells.

## Supplementary Information

Supplementary Table 1.

Supplementary Table 2.

## Data Availability

The data that support the findings of this study are available from the corresponding author upon reasonable request.

## Abbreviations

IS: Ischemic stroke
HRG: Hypoxia-related gene
NK: Natural killer
BBB: Blood–brain barrier
GEO: Gene Expression Omnibus
MSigDB: Molecular signature database
DEGs: Differentially expressed genes
LASSO: Least absolute shrinkage and selection operator
ROC: Receiver operating characteristic
BP: Biological process
CC: Cellular component
MF: Molecular function
KEGG: Kyoto Encyclopedia of Genes and Genomes
ROS: Reactive oxygen species
D.C.s: Dendritic cells
MACO: Middle cerebral artery occlusion
Tregs: Regulatory T cells
TIA: Transient ischemic attack
